# Coronary Artery-Bypass-Graft Surgery Increases the Plasma Concentration of Exosomes Carrying a Cargo of Cardiac MicroRNAs: An Example of Exosome Trafficking Out of the Human Heart with Potential for Cardiac Biomarker Discovery

**DOI:** 10.1371/journal.pone.0154274

**Published:** 2016-04-29

**Authors:** Costanza Emanueli, Andrew I. U. Shearn, Abas Laftah, Francesca Fiorentino, Barnaby C. Reeves, Cristina Beltrami, Andrew Mumford, Aled Clayton, Mark Gurney, Saran Shantikumar, Gianni D. Angelini

**Affiliations:** 1 Bristol Heart Institute, University of Bristol, Bristol, United Kingdom; 2 National Heart and Lung Institute, Imperial College London, London, United Kingdom; 3 Institute of Cancer & Genetics, University of Cardiff, Cardiff, United Kingdom; Northwestern University, UNITED STATES

## Abstract

**Introduction:**

Exosome nanoparticles carry a composite cargo, including microRNAs (miRs). Cultured cardiovascular cells release miR-containing exosomes. The exosomal trafficking of miRNAs from the heart is largely unexplored. Working on clinical samples from coronary-artery by-pass graft (CABG) surgery, we investigated if: 1) exosomes containing cardiac miRs and hence putatively released by cardiac cells increase in the circulation after surgery; 2) circulating exosomes and exosomal cardiac miRs correlate with cardiac troponin (cTn), the current “gold standard” surrogate biomarker of myocardial damage.

**Methods and Results:**

The concentration of exosome-sized nanoparticles was determined in serial plasma samples. Cardiac-expressed (miR-1, miR-24, miR-133a/b, miR-208a/b, miR-210), non-cardiovascular (miR-122) and quality control miRs were measured in whole plasma and in plasma exosomes. Linear regression analyses were employed to establish the extent to which the circulating individual miRs, exosomes and exosomal cardiac miR correlated with cTn-I. Cardiac-expressed miRs and the nanoparticle number increased in the plasma on completion of surgery for up to 48 hours. The exosomal concentration of cardiac miRs also increased after CABG. Cardiac miRs in the whole plasma did not correlate significantly with cTn-I. By contrast cTn-I was positively correlated with the plasma exosome level and the exosomal cardiac miRs.

**Conclusions:**

The plasma concentrations of exosomes and their cargo of cardiac miRs increased in patients undergoing CABG and were positively correlated with hs-cTnI. These data provide evidence that CABG induces the trafficking of exosomes from the heart to the peripheral circulation. Future studies are necessary to investigate the potential of circulating exosomes as clinical biomarkers in cardiac patients.

## Introduction

Extracellular microRNAs (miRs) are resilient to degradation and thus are long-lived in biological fluids and measurable in both freshly collected and banked clinical samples[1]. miRs, together with other acid nucleic molecules, proteins, and lipids can be released from different cell types as the cargo in extracellular vesicles (EVs), including apoptotic bodies, microparticles and exosomes[1, 2]. Exosomes are the smallest (30 to 100 nm in diameter) endogenous EVs identified so far[2]. They are released through the exocytosis of multivesicular bodies (MVBs)[3]. In contrast to other EVs, exosomes are thought to be released by living cells through an active process, but not by lysed or apoptotic cells[2, 4–6]. Cardiac and vascular cells have been shown to produce exosomes *in vitro*. Observational data indicate that the abundance of released exosomes and their molecular cargo differ in response to environmental perturbations, including hypoxia[7, 8], changes in D-glucose concentration[8–10], and stimulation with Angiotensin II[11]. *In vitro*, exosomes have been shown to transfer biologically active components of their cargo into “recipient” cells, thus playing a role in cell-to-cell communication[10, 12]. It is of note that the full cascades of the molecular mechanisms regulating exosome formation, release and uptake under homeostatic conditions or after environmental perturbations are not clear, however they are starting to be deciphered. For example, integrins expressed on the membrane of tumour cell-derived exosomes have been recently shown to determine organotropic metastasis by guiding the preferential cross talk between distant and specific cell types[13, 14]. In the cardiovascular area, equivalent mechanistic studies are still lacking. Additionally, technical limitations linked to their nanoscale size make the exosome trafficking *in vivo* difficult to explore. Nonetheless, it is possible to track nanoparticles in biological fluids,[2] as we have done in our study. Additionally, several approaches, including the column-based separation method employed here, allow the enriching of exosomes from biological fluids in a form that is compatible with functional and expressional and analyses, including of exosomal miRs[2].

Interventional cardiologists have already provided evidence that cardiac expressed miRs (miR-1, miR-133a, miR-133b, miR-208a, miR-208b, and miR-499) increase in the blood acutely following a myocardial infarction (MI) and some of these studies have additionally scrutinized the diagnostic potential of miRs by comparisons with cTns[15–17]. We reasoned that additional miRs could be regulated by cardiac ischemia and ischemia reperfusion and hence found expressionally altered in the peripheral circulation. These include miR-210, which is also commonly identified as “hypoxia miR” is expressed in the heart and it increases under hypoxic conditions, including in cardiac myocytes[18–21] and miR-24, which is upregulated in the myocardium and its endothelial cells, but downregulated in cardiac myocytes following the experimental induction of an ischemic event in mice[22, 23]. In the setting of cardiac surgery, miR-1 was found increased in the blood at 1h and 24h after mitral valve replacement[24]. Similarly, miR-1 and miR-208a increased at 45 min after aortic clamping in patients undergoing combined mitral and aortic valve replacement[25]. However, despite of the initial enthusiasm, miRs have not yet had an impact on clinical practice in the diagnosis of ischemic heart damage. Moreover, whether those myocardial ischemia-responsive miRs were carried in the peripheral blood by EVs has not been investigated.

Coronary-artery-by-pass-graft (CABG) surgery using cardiopulmonary bypass (CPB) and myocardial cardioplegic arrest is one of the most commonly performed operations in the world (bluebook.scts.org -Blue Book Online-Society for Cardiothoracic Surgery). CABG is intrinsically associated with variable degrees of myocardial damage related to the use of CPB and the ischemia-reperfusion insult of the cardioplegic arrest. We reasoned that this clinical setting would allow the determination of whether cardiac exosomes are released in response to myocardial injury and if this is sufficient to significantly affect the abundance and cargo of the pool of exosomes circulating in the peripheral blood. Moreover, the potential of circulating exosomes as novel biomarker of heart injury could be assessed by correlating circulating exosomes and elements of their molecular cargo with cardiac troponins (cTns), the “gold standard” surrogate biomarkers of myocardial damage[26]. CTns are commonly employed in cardiac surgery practice and in clinical studies. While intra-operatory myocardial infarct (MI) happens in a minority of cardiac surgery patients, any operation on CPB is inevitably associated with some level of cardiac damage due to ischemia/reperfusion and the circulating cTns levels are elevated after the operation[27]. Highly sensitive assays have shortened the time by when cTns pick up in the blood from 6 hours to 1 hour after the reperfusion. However, not all diagnostic laboratories are able to measure hs-cTns and the search for novel early biomarkers of myocardial damage measured in the circulating blood is an important goal with multiple translational applications.

Here, working on banked plasma samples from two prospective observational clinical studies, we have sequentially investigated the changes in the plasma concentrations of cardiac miRs, exosomes and of the exosomal cardiac-expressed miRs in CABG patients.

## Materials and Methods

### Clinical Studies

The clinical studies were approved by UK National Health Service research ethics committees and were conducted in accordance with the principles of the International Conference on Harmonisation-Good Clinical Practice under the oversight of University Hospitals Bristol National Health Service Foundation Trust (COPTIC) and the Imperial College Health Service Foundation Trust (ARCADIA). All patients provided written informed consent.

COPTIC (*Coagulation and Platelet Function Testing in Cardiac Surgery*), a registered (ISRCTN20778544) UK National Institute of Health Research (NIHR)-funded single-centre prospective observational study, recruited 2,427 patients undergoing cardiac surgery at the Bristol Heart Institute. It has collected citrate plasma (from the arterial blood line) before (prior to chest opening) and on completion of surgery (before chest closure). The former sample (pre-operation: pre-op) is taken before heparin administration and the latter (post-operation: post-op) within 30 minutes of reversal of heparin anticoagulation by protamine sulfate, when the patient is still in the operating theatre. The COPTIC research protocol was amended and ethically approved to perform microRNA (miR) and exosome analyses. In this study, we have used pre-op and post-op samples (banked at a temperature of between -70°C and -80°C) from a subgroups of male non-diabetic patients (n = 15), aged less than 75 years undergoing coronary artery bypass graft (CABG) surgery using cardiopulmonary bypass (CPB). See Table 1 for patients’ characteristics.

**Table 1:** Characteristics of the COPTIC- coronary artery bypass graft (CABG) patients used in the study.

Characteristic		Total n = 15	
Age (years; median, IQR)		67.8	(63.2, 71.6)
Sex (males; n, %)		15/15	100%
Previous MI (n, %)		10/15	67%
Hypertension (n, %)		13/15	87%
Diabetes (n, %)		0/15	0%
eGFR (median, IQR)		79.9	(66.5, 108.3)
BMI (kg/m^2^; median, IQR)		26.3	(24.3, 28.4)
*Logistic Euroscore (median, IQR)		2.2	(1.5, 4.0)
NYHA class (n, %)	Class 1	7/15	47%
	Class 2	6/15	40%
	Class 3	2/15	13%
CCS class (n, %)	Class 0	2/15	13%
	Class 1	4/15	27%
	Class 2	5/15	33%
	Class 3	0/15	0%
	Class 4	4/15	27%
Operative priority (n, %)	elective	8/15	53%
	urgent	7/15	47%
CPB time (minutes; median, IQR)		70	(50, 93)
Cross-clamp time (minutes; median, IQR)		39	(30, 48)
Number of grafts (n, %)	2	6/15	40.0%
	3	7/15	46.7%
	4	2/15	13.3%
In-hospital mortality		0/15	0.0%

* Logistic Euroscore is missing for 1 patient

ARCADIA (*Association of non-coding RNAs with Coronary Artery Disease and type 2 Diabetes*) (REC 13/LO/1687) is a prospective observational study conducted at the Bristol Heart Institute and at the London Hammersmith Hospital. ARCADIA has been specifically designed to carry out analyses of non-coding RNAs (ncRNA), including miRs and extracellular vesicles (EVs), including exosomes, in patients undergoing first time CABG using CPB and cold blood cardioplegic arrest. Blood samples are taken at 4 time points: 1) in the anaesthetic room; 2) during surgery before establishment of CPB, and at 3) 24h and 4) 48h after the end of the operation. For this study, we used anonymized citrate plasma (for miR and exosome analyses) and serum (for hs-cTn-I) samples from 6 male non-diabetic CABG patients previously stored at -80°C. See Table 2 for patients’ characteristics.

**Table 2:** Characteristics of the ARCADIA- coronary artery bypass graft (CABG) patients used in the study.

Characteristic		Total n = 6	
Age (years; median, IQR)		67.5	(63.0, 74.0)
Sex (males; n, %)		6/6	100%
Previous MI (n, %)		3/6	50%
Hypertension (n, %)		5/6	83%
Diabetes (n, %)		0/6	0%
eGFR (median, IQR)		81	(75, 90)
BMI (kg/m^2^; median, IQR)		28.2	(26.8, 30.4)
Logistic Euroscore (median, IQR)		2.5	(1.5, 4.0)
NYHA class (n, %)	Class 1	5/6	83%
	Class 2	1/6	17%
CCS class (n, %)	Class 0	2/6	33%
	Class 1	2/6	33%
	Class 2	1/6	17%
	Class 3	1/6	17%
	Class 4	0/6	0%
Operative priority (n, %)	Elective	1/6	17%
	Urgent	5/6	83%
CPB time (minutes; median, IQR)		76.5	(69, 90)
Cross-clamp time (minutes; median, IQR)		44	(37, 61)
Number of grafts (n, %)	2	2/6	33%
	3	3/6	50%
	4	1/6	17%
In-hospital mortality (n, %)		0/6	0%

### Methods Overview

Anonymised raw data that were analysed are available in the S1 Data Dataset used for analyses.

The plasma exosome concentration was analysed using a Nanoparticle Tracking Analysis (NTA) machine (NanoSight). The plasma concentration of particles of a size (30 to 100nm) typical of exosomes[2] was determined together with the particle distribution by size.

Using a column-based system, exosomes were enriched from the ARCADIA patients’ plasma.

S1 Table presents the individual miRs measured (RT-PCR) in plasma and plasma exosomes, with PCR primers and reasons for their inclusion in this study.

Hs-cTn-I was measured in serial ARCADIA serum samples (ARCHITECT STAT, Abbott). The COPTIC samples were analysed with a non-hs cTn-I ELISA (Sigma) since the available hs-cTn-1 assay (and of other hs-cTn assays) is unsuitable for citrate plasma samples.

#### Statistical analyses

For the COPTIC samples, regression analysis was used to investigate differences between pre- and post-cardiac surgery levels of extracellular miRs and exosomes. Results are illustrated graphically as scatter plots on a log scale with pre-surgery against post-surgery levels for either each miR or exosome concentration. Summary statistics are presented as mean difference, standard error (SE) and confidence interval for both pre- and post-surgery levels. For the ARCADIA samples, comparisons over each of the four time points were performed using repeated measures ANOVA with the *post hoc* Tukey’s test. Data are presented as the mean ± standard error of the mean (SEM). Comparisons between individual miR expression levels, exosome concentrations and log_e_ hs-cTn-I concentrations were made using simple linear regression, with the regression coefficient (± SE) reported in each case to indicate the predictive power of the explanatory variable. Significance levels were determined using the F test, and data from all time points were analysed simultaneously. Statistical analyses were performed using STATA (v13, STATA Corp., TX, USA) and Prism (v6, GraphPad Software, CA, USA). The level of significance was taken as p<0.05.

### Detailed Methods

#### Exosome analyses

The plasma nanoparticle concentration was analysed using a Nanoparticle Tracking Analysis (NTA) system (NanoSight, Amesbury, UK). One μL of neat plasma was diluted with ultra-clean, sterile water to obtain a concentration of particles suitable to be read on the machine in a 1mL sample, according to the manufacturer’s guidelines. This was then passed through a flow cell at a constant flow rate using a syringe driver, where a laser beam was shone through the stream of particles. Once the temperature of the flow cell had stabilised at 25°C, 6 individual videos of 30 sec duration were recorded consecutively, with a 5 sec delay in between each. These videos were then processed using the NTA software, which uses the Stokes-Einstein Equation to determine the size of the particles from their Brownian motion[2]. The data were elaborated to be presented as the average of these 6 videos taken in one run. The equipment used determines the size of the particles in the solution up to around 1–2μm and a histogram of the concentration of particles per mL for each nanometre size can be plotted with the resulting data[28]. The plasma concentration of nanoparticles within the exosome size range (30–100nm)[2] was determined. In the ARCADIA sample set, the average particle distribution by size was also determined. NTA was also used on exosome preparations to validate their particle sizes.

#### Exosome enrichment from plasma samples

Using a column-based system, Exo-spin Mini-Columns (Cell Guidance Systems, Cambridge, UK), exosomes were enriched from the ARCADIA patients’ plasma. Briefly, 100 μL of plasma prepared as described above was further centrifuged at 17,000 *g* for 30 min at 4°C to remove any remaining cell debris. The supernatant was transferred to a fresh tube and 5μL thrombin (500 U/mL) per 0.5 mL plasma was added to each sample, to remove the fibrin proteins. The samples were incubated at RT for 15 minutes while mixing, then centrifuged at 10,000 *g* for 5 min at RT. The supernatant was then passed through a sterile a 0.22μm filter (Merck Millipore, Cork, Ireland) into a fresh tube. A sample volume of 100μL was applied to the top of the column and centrifuged at 500 g for 60 sec. After discarding the elute, 200μL of PBS was applied to the top of the column, the sample was centrifuge at 500 g for 60 seconds and the elute containing purified exosomes was stored at -20°C until required.

#### Transmission Electron Microscopy and Western blot analyses on exosome preparations

Exosomes from Patient 6 of the ARCADIA cohort were extracted using the ExoSpin columns as described above. Negative staining and TEM was carried out using an adapted, previously described protocol[29]. Briefly, 3μL of the exosome preparation was incubated on a Pioloform carbon-coated grid at RT for 10 minutes. The grid was then washed on a droplet of distilled water for 10 minutes. Finally, the grid was incubated in a solution of 1.8% methylcellulose and 0.3% uranyl acetate for 5 minutes on ice. The grid was then removed with a loop, the excess liquid drained off using a Whatman 1 filter and allowed to air dry. The grids were imaged using a Tecnai12 120 kV BioTwin Spirit transmission electron microscope (FEI Company, Eindhoven, Netherlands) equipped with a bottom-mounted Eagle CCD camera (FEI).

Western blot analyses for exosome markers were performed on the plasma exosomal fractions extracted as above. Exosome preparations were lysed with RIPA buffer (Santa Cruz Biotechnology) with added protease inhibitor cocktail. Samples were centrifuged at 14,000 *g* for 15 min at 4°C and the supernatant fractions were used for Western blot. The protein content was measured using MicroBCA protein assay (Thermo Scientific, Hemel Hempstead, UK). Primary and secondary western blot antibodies are shown in S2 Table.

#### MicroRNA analyses

All the miRs measured in COPTIC and ARCADIA, together with their sequences, reasons for inclusion in the analyses (with literature references) and the miR assay codes are summarized in S1 Table. In the COPTIC plasma samples, we measured a selection of miRs known to be enriched in the myocardium (miR-1, miR-133a, miR-133b, miR-208a, miR-208b) and regulated by ischemia in cardiovascular cells and/or patients with an acute myocardial infarction (miR-1, miR-24, miR-92a, miR-126, miR-133a, miR-133b, miR-208a, miR-208b, miR-210) (see in S1 Table). MiR-451 was measured as a quality control against haemolysis since it is enriched in red blood cells and miR-23a because it is supposedly stably expressed in plasma[30]. Moreover, miR-233 was measured because it is enriched in platelets[31], its circulating level are correlated with platelet reactivity index in patients requiring revascularization for coronary artery disease[32] and there is a platelet response to cardiac surgery[33]. We did not measure exosomal miRs in COPTIC due to the low volume of available plasma making it insufficient to prepare exosome fractions suitable for miR analyses. In ARCADIA, a selection of the aforementioned miRs (miR-1, miR-24, miR-133a, miR-133b, miR-210) together with the negative control (for cardiac expression) liver-specific-miR-122 were measured both in whole plasma and its exosomal fraction. In preparation for PCR analyses, RNA was isolated using the miRNeasy kit (Qiagen, Valencia, CA) according to the manufacturer’s instructions. For RNA extraction from plasma, 200 μL of exosome sample was used with 1mL QIAzol. Cel-miR-39 was spiked-in (10μl of a 5fmol/μL stock) to normalize for RNA extraction efficiency[34]. In ARCADIA (where the second time point was collected in heparinized patients), all the RNA samples were treated with heparinase-I (2 U/ μl- Sigma-Aldrich, catalogue number: H2519). S3 Table provides evidence that this treatment does not affect PCR analyses in plasma derived from non-heparinized subject, while it can “resuscitate” miR detection by PCR in plasma contaminated with heparin[35]. All COPTIC and ARCADIA samples were treated with RNase inhibitor (50 U/ μl). The reverse transcription reaction for each individual miR was performed using the TaqMan miR Reverse Transcription Kit and miR-specific stem-loop primers (Life Technologies, Paisley, UK), and quantitative PCR (qPCR) was then performed using 2x Universal PCR Master Mix with No AmpErase UNG (Life Technologies, Paisley, UK). MiR-specific stem-loop primer identification numbers are presented in the S1 Table. Expression of each miR was normalised to cel-miR-39. Each PCR reaction was performed in triplicate and data were calculated using the 2−ddCt method.

#### Cardiac troponin-I measurement

High Sensitive (hs) cardiac Troponin-I (cTn-I) (hs-cTn-I) was measured in serial ARCADIA serum samples by using the ARCHITECT STAT High Sensitive Troponin-I assay (Abbott). In the COPTIC samples, we measured cTn-I by ELISA (Sigma), because the ARCHITECT or other kits for either hs-cTn-I or hs-cTn-T do not work on plasma citrate samples.

## Results

### CABG is associated with a rapid increase in plasma cardiac miRs

COPTIC (ISRCTN20778544) was a prospective observational study in patients undergoing heart surgery in which citrate plasma samples were collected at two time points: 1) in the anaesthetic room before surgery and 2) post-CPB immediately after heparin reversal before chest closure. Table 1 summarizes the characteristics of the COPTIC patients who contributed samples to in this study. The COPTIC biobank offered the possibility for studying early responses after CABG using CPB and cold blood cardioplegic arrest (a subgroup of the COPTIC population). A set of miRs that are reportedly expressed by cardiac myocytes and upregulated by ischemia in the heart (see the S1 Table) increased early after CABG (Table 3 and S1 Fig). Moreover, in line with the known CBP-induced platelet activation, CABG increased the plasma level of platelet-enriched miR-223. By contrast, the vascular expressed miR-92a and miR-126 and the “quality control” miR-23a (supposedly stably expressed in plasma)[30] and miR-451 (used as control against plasma sample haemolysis since it is enriched in red blood cells)[36] were unaffected by CABG.

**Table 3:** Summary of the mean differences between miR levels before and after CABG with corresponding standard errors (SE) and levels of significance.

miRNA (No)	Mean difference between the natural logarithm of the pre and post levels (SE)	95% Confidence Interval	P value
miR-1	3.20 (0.57)	1.986–4.420	p < 0.001
miR-23a	-0.27 (0.34)	-1.001–0.466	p = 0.4468
miR-24	0.93 (0.28)	0.330–1.521	p = 0.0049
miR-92a	0.83 (0.29)	0.198–1.454	p = 0.159
miR-126	0.51 (0.25)	-0.025–1.036	p = 0.0601
miR-133a	3.11 (0.37)	2.326–3.902	p < 0.001
miR-133b	1.36 (0.59)	0.093–2.636	p = 0.0372
miR-208a	1.31 (0.75)	-0.298–2.926	p = 0.1023
miR-208b	0.67 (0.81)	-1.060–2.399	p = 0.4205
miR-210	1.18 (0.28)	0.584–1.776	p < 0.001
miR-223	1.32 (0.18)	0.944–1.702	p < 0.001
miR-451	0.05 (0.27)	-0.532–0.635	p = 0.8530

### CABG is associated with a rapid increase in plasma exosomes

**Fig 1:**
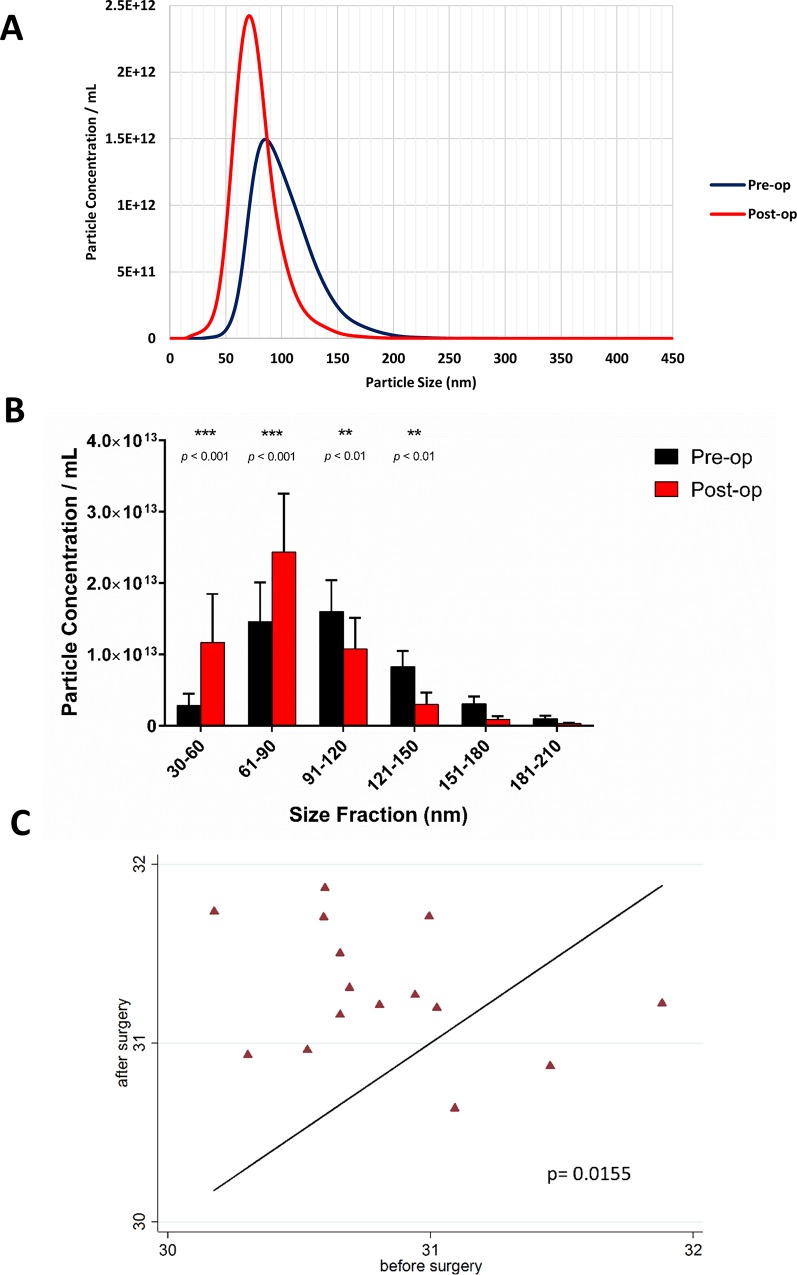
**Early changes in plasma exosome-sized nanoparticle concentration after CABG: A**) Average particle distributions by size in the serial plasma samples taken from CABG patients (n = 15) immediately before (pre-operation = pre-op) and at termination of surgery, before chest closure (post-operation = post-op. **B**) Graph showing the average size distribution of particles from all of the pre- and post-operative blood samples as determined by Nanoparticle Tracking Analysis. Data are shown as mean + SEM; repeated measures ANOVA with *post hoc* Tukey’s test. **C**) Regression analyses of changes in plasma exosome concentration (here identified as particles between 30 and 100 nm in size) per mL of plasma after the operation. The graph shows pre- vs. post-operation exosome levels (the solid line represents no change between the pre and post levels). Data were analysed by linear regression method. The P values of the different analyses are presented as part of the graphs in panels **B** and **C**.

In the COPTIC samples, plasma circulating nanoparticles of the size of exosomes increased immediately after completion of CABG, when the patients were still in the operating theatre (Fig 1A). The analysis of the size distribution of EVs provided evidence that CABG increased the density of vesicles of the size of exosomes (30 to 100 nm circa), but it did not affect the amount of larger particles (above 120 nm) (Fig 1B). The concentration of exosomes increased by 55% compared to before surgery (mean difference in the natural logarithm of the exosome concentration was 0.46±0.17 (95% CI [0.10, 0.82]), p = 0.0155 (Fig 1C). S2 Fig shows the early post-CPB changes in circulating exosomes in 6 randomly selected patients out of the 15 investigated.

### Time course of changes in circulating cardiac miRs, exosomes, and exosomal miRs induced by CABG

**Fig 2:**
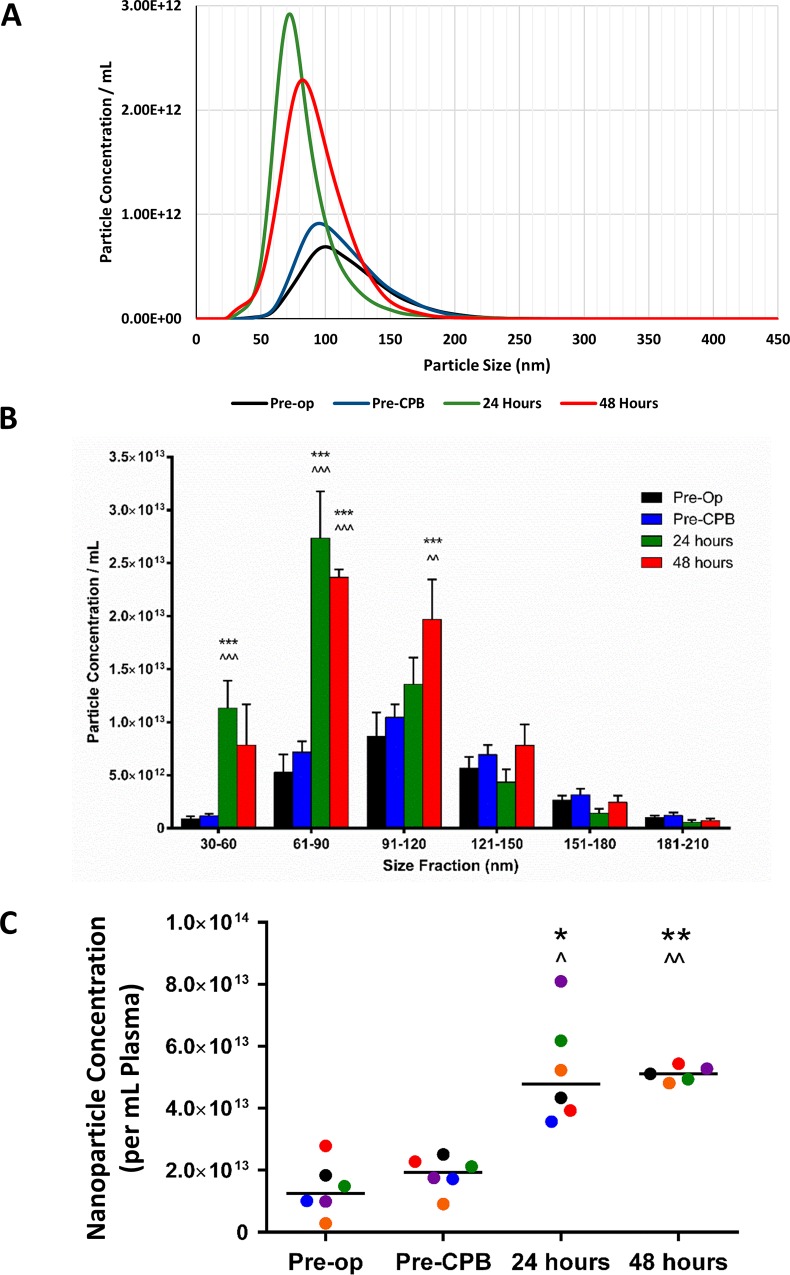
**Time-course of plasma exosome-sized nanoparticle concentration in CABG patients: A**) Average particle distributions by size in the serial plasma samples taken from CABG patients (n = 6) immediately before (pre-operation = pre-op), during the operation before starting coronary grafting with the patient on cardiopulmonary-by-pass (pre-CPB) and at 24h and 48h after the operation. **B**) Average particle distributions by size in the serial plasma samples (n = 6). Plasma was prepared from blood collected immediately before (pre-operation, pre-op); during the operation, before starting coronary grafting with the patient on cardiopulmonary by-pass (CBP) (pre-CPB) and at 24h and 48h from completion of surgery. Data are shown as mean + SEM. *** *p* < 0.001 *vs*. pre-op; ^^ *p* < 0.01, ^^^ *p* < 0.001 *vs*. pre-CPB; repeated measures ANOVA with *post hoc* Tukey’s test. **C**) Plasma exosome concentration (here identified as particles between 30 and 100 nm in size) per mL of plasma at each of the four time points. Results presented as individual data points and median, with each individual patient in a unique colour. * *P*< 0.05, ** *p* < 0.01 *vs*. pre-op; ^ *p* < 0.05, ^^ *p* < 0.01 *vs*. pre-CPB; repeated-measures ANOVA with *post hoc* Tukey’s test.

We next investigated the time-course of plasma miRs and exosomes response to cardiac surgery and changes in the exosomal miRs cargo. These tasks were developed using samples from a subgroup of CABG patients (see Table 2 for the patients’ characteristics) belonging to a different cardiac surgery cohort: ARCADIA (REC 13/LO/1687), an ongoing prospective observational study. Citrate plasma and serum samples were collected at four time points: 1) in the anaesthetic room before surgery; 2) during surgery before establishment of CPB, 3) at 24h post-CPB and 4) 48h post-CPB. As shown in Fig 2A to 2C, there were no changes in plasma exosome concentration during the operation, before the CPB initiation. By contrast, plasma exosomes were elevated at 24h and 48h after the operation. These changes followed similar patterns in individual patients (S3 Fig).

**Fig 3:**
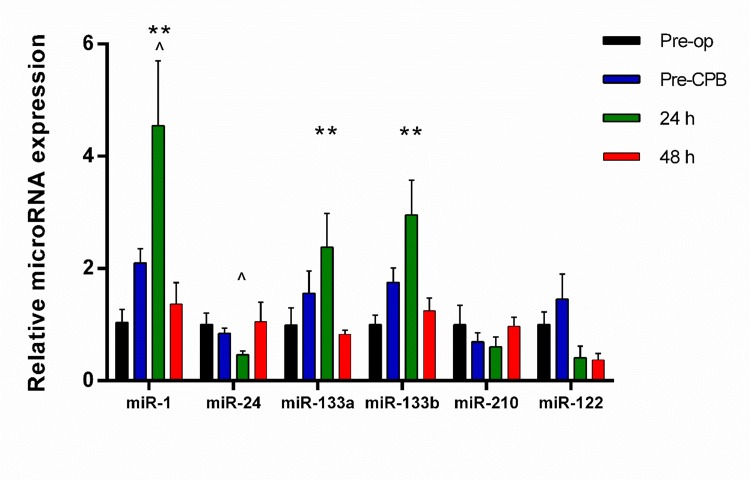
Time-course of miR changes in the total plasma in CABG patients: Expression of each miR in the total plasma relative to pre-op levels. Results presented as mean + SEM. ** *P* < 0.01 *vs*. pre-op; ^ *p* < 0.05 *vs*. pre-CPB; repeated measures ANOVA with *post hoc* Tukey’s test.

As shown in Fig 3 and Table A in S4 Table, the concentrations of miR-1, miR-133a and miR-133b increased in the whole plasma at 24h post-surgery. By contrast, the concentration of miR-24 did not. No other miR expressional changes in the measured were observed. As shown in the S1 Table, the numbers of miRs measured in ARCADIA were reduced in comparisons to COPTIC.

**Fig 4:**
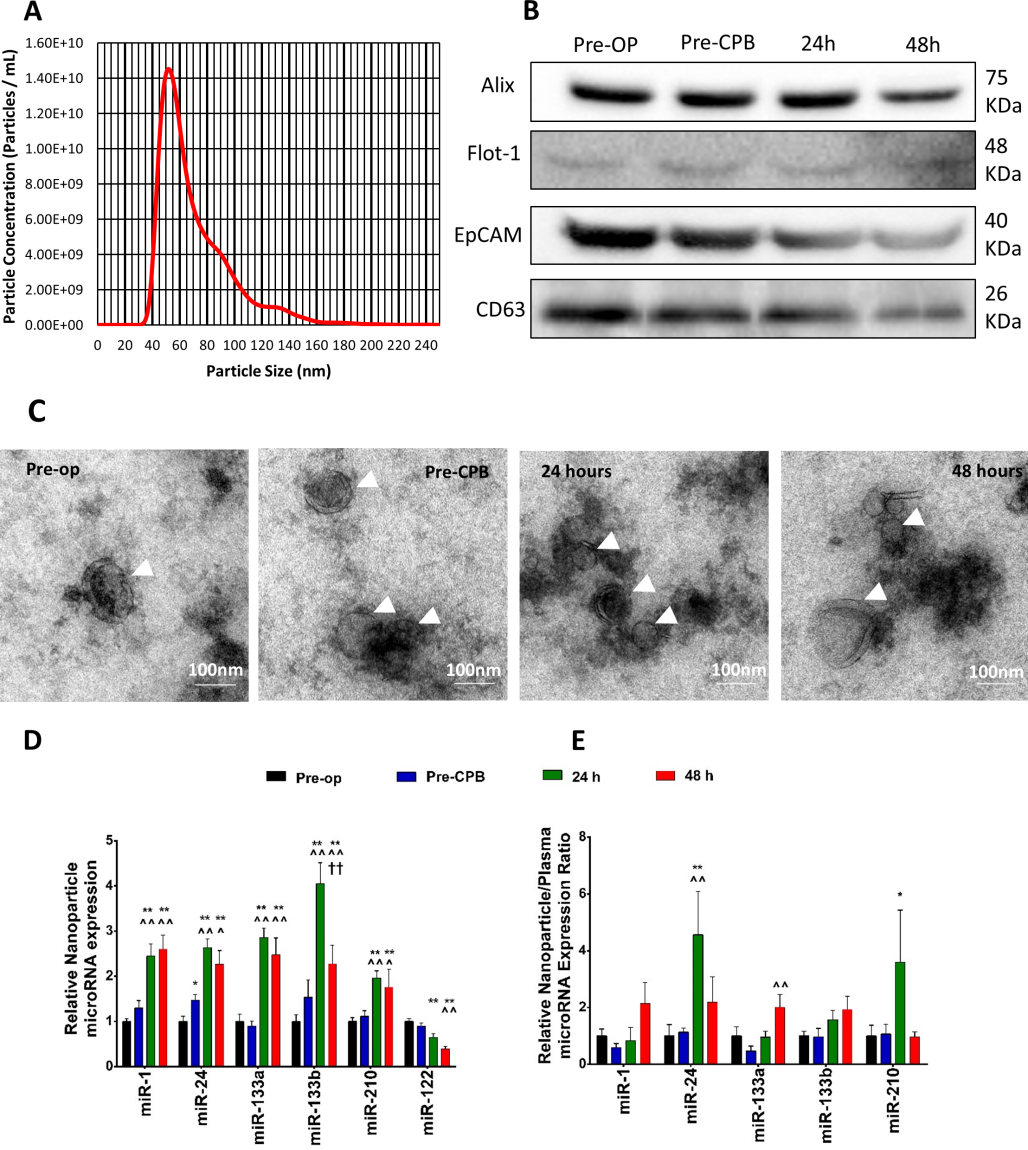
Time-course of miR changes in the total plasma and plasma exosomal fraction associated with CABG, and exosomal miR/total plasma miR ratios at different time-points: Validation of exosome enrichment from the plasma of CABG patients. (**A**) Analysis of the exosome preparation by Nanoparticle Tracking Analysis. Data shown are the average from 6 individual patients (30-second videos recorded concurrently and in one run). (**B**) Western blot analyses for exosome antigens Alix, Flot-1, EpCAM and CD63. The results on exosome fractions prepared from one patient’s plasma collected at each of the time points used in ARCADIA are shown **(C)** Electron micrographs of exosomes extracted from plasma from the same patient as in (B). Expression of each studied miR in the plasma exosome fraction (**D**), and exosome/plasma miR expression ratio (**E**) relative to pre-op levels. Results presented as mean + SEM. ** *P* < 0.01 *vs*. pre-op; ^ *p* < 0.05 and ^^ *p* < 0.01 *vs*. pre-CPB; †† *p* < 0.01 *vs*. 24 hours; repeated measures ANOVA with *post hoc* Tukey’s test.

Next, we prepared an exosome-enriched fraction from aliquots of the same plasma samples employed for the analyses of above. We assessed the quality of our preparations. NTA showed that our preparation contained vesicles of the size (30 to 100 nm) of exosomes (Fig 4A). Additionally, the preparations expressed a panel of exosome markers (Alix, Flot-1, EpCAM, CD63) (Fig 4B) and TEM demonstrated the presence of particles of size and morphology (double membrane) typical of exosomes (Fig 4C).

Interestingly, the pattern of miR changes associated with CABG was different when miRs were measured in the plasma exosomal fraction rather than in whole plasma (Fig 4C and Table B in S4 Table). In detail, concentrations of exosomal miR-1, miR-24, miR-133a and miR-133b all increased at both 24h and 48h post-surgery, while some of these changes were not detected in the whole plasma (see above). The calculation of individual miR concentration ratios between whole plasma and plasma exosomes showed that miR-24 was especially enriched in the exosomal fraction after CABG. The miR-210 exosome/whole plasma concentration ratio also increased (Fig 4D) at 24h post-CPB.

### Correlations between exosomes, miRs, exosomal miRs and cTn-I

**Fig 5:**
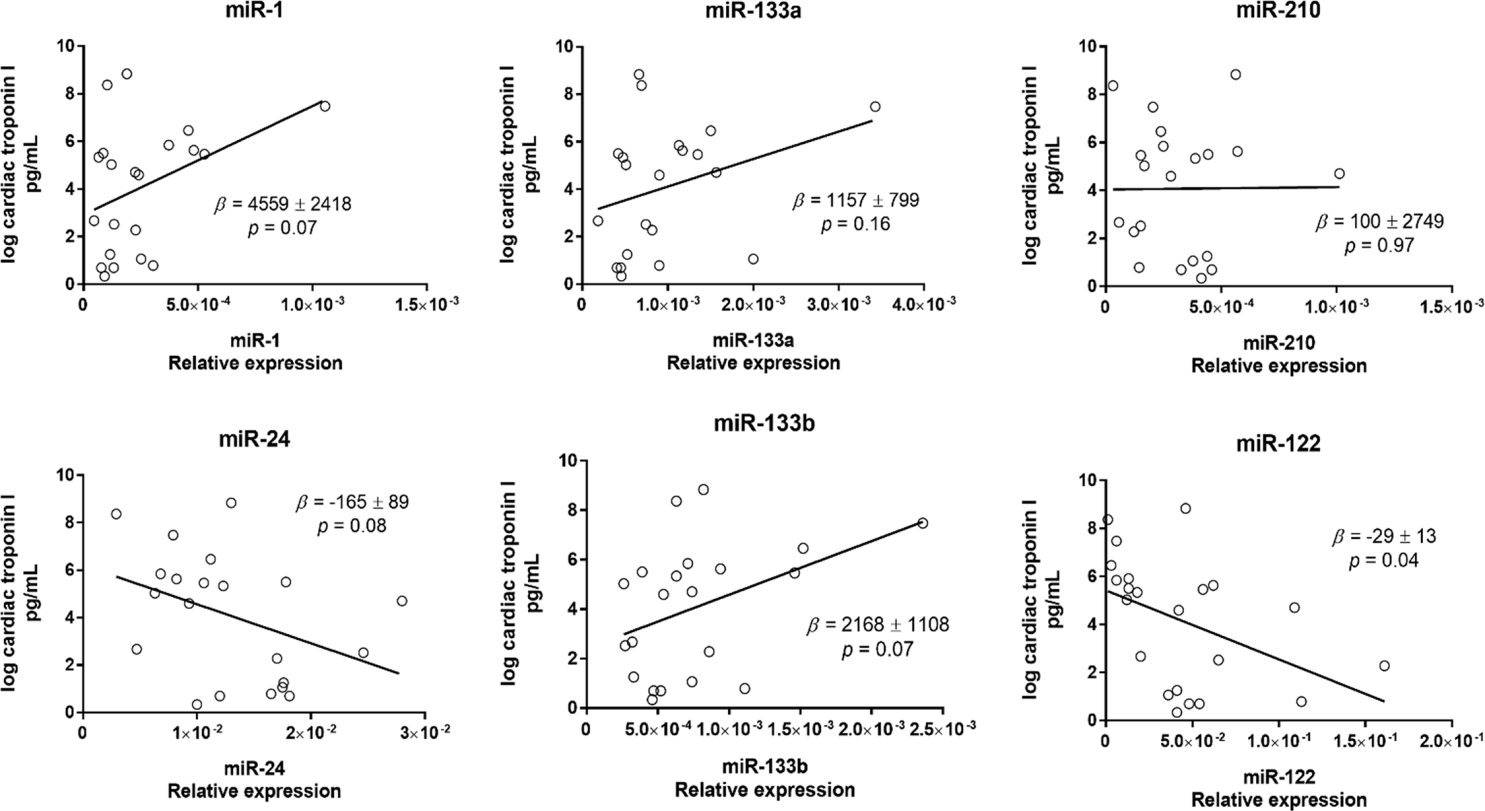
Linear regression of relative expression of plasma concentration of individual miRs and cTn-I. All time-points were included. The regression coefficients (β ± standard error) and P values are indicated in the plots.

**Fig 6:**
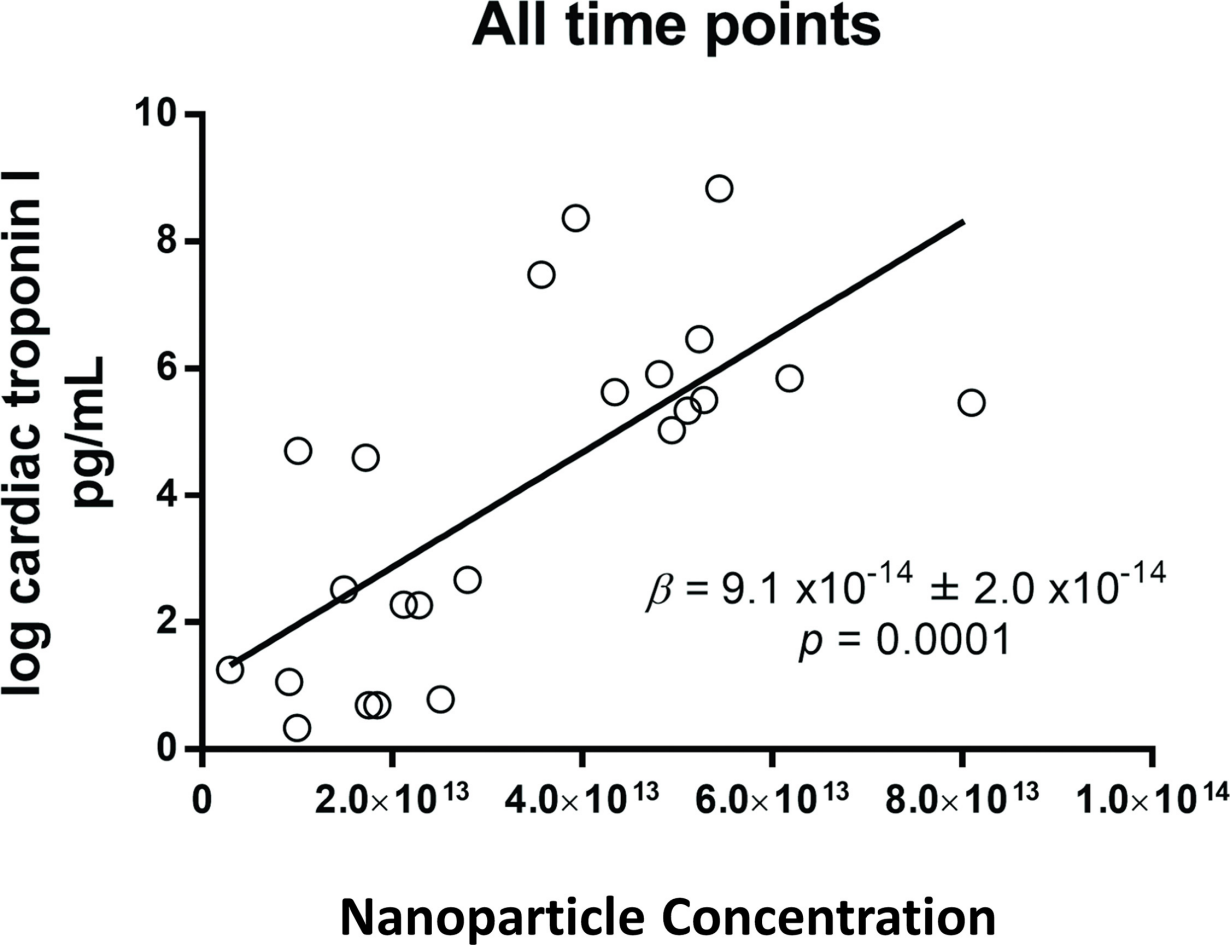
Linear regression of exosome plasma concentration versus cardiac troponin I (cTn-I). All time-points were included. The regression coefficients (β ± standard error) and P values are indicated in the plots.

**Fig 7:**
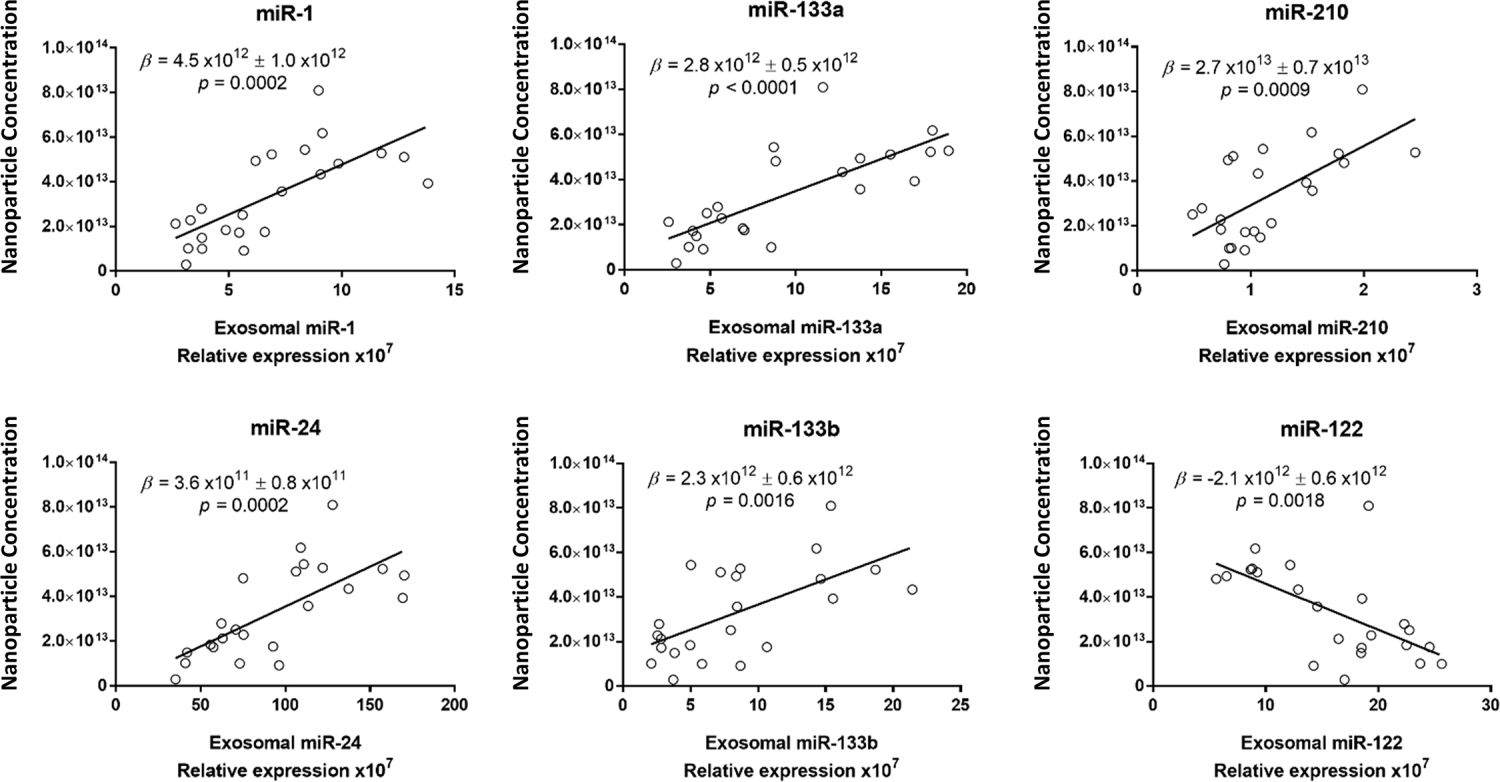
Linear regression of each exosomal miR and plasma exosome concentration. All time-points were included. The regression coefficients (β ± standard error) and P values are indicated in the plots.

**Fig 8:**
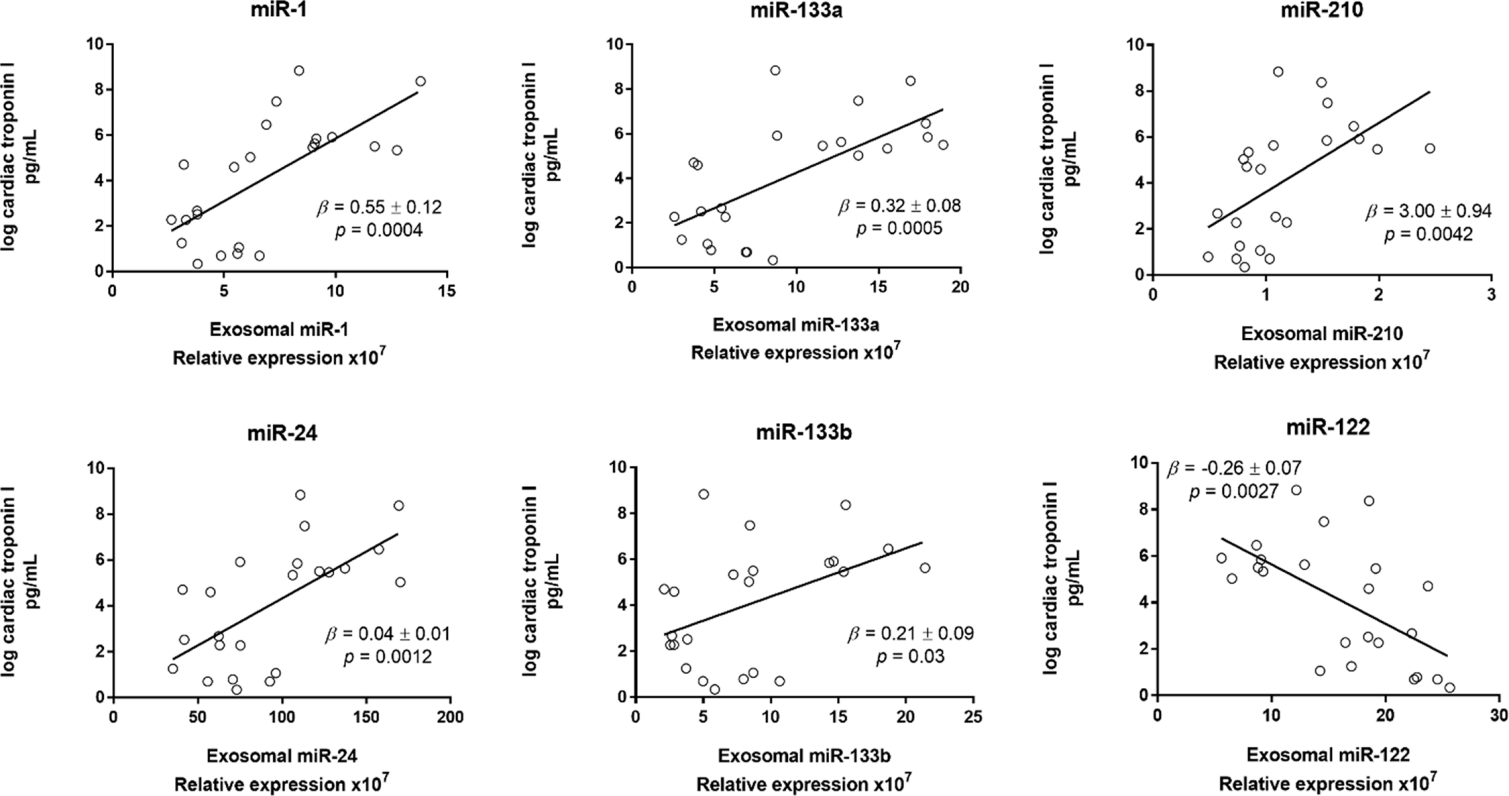
Linear regression of relative expression of each exosomal miR and cTn-I. All time-points were included. The regression coefficients (β ± standard error) and P values are indicated in the plots.

CTn-I was unchanged at completion of surgery (data not shown). However, the hs-cTn-I concentration increased at 24h and 48h post-CPB (S5 Table and S4 Fig). The whole plasma concentration of individual miRs was not correlated with hs-cTn-I (Fig 5). By contrast, the plasma exosome concentration was strongly positively correlated with hs-cTn-I (β = 9.1x10^-14^ ± 2.0x10^-14^, P<0.0001) (Fig 6). The concentration of total exosomes was positively correlated with the concentration of exosomal miR-1, miR-133a, miR-24, miR-210 and miR-133b (Fig 7). Finally, the exosomal concentrations of miR-1, miR-133a, miR-24, miR-210 and miR-133b were strongly positively correlated with cTn-I (Fig 8).

## Discussion

This study provides novel evidence that: 1) the concentration of cardiac-enriched, ischemia-responsive miRs and of nanoparticles of exosome size increases in the plasma early after CABG; 2) miRs of possible cardiac origin circulate as part of exosomes and in non-exosomal plasma fractions, with the exosome to whole plasma concentration ratios of individual miRs differently affected by surgery; 3) the concentrations of exosomes and of exosomal cardiac miRs after CABG are positively correlated with the concentration of hs-cTn-I; 4). Following CABG, exosomes increased in the peripheral circulation earlier than cardiac troponins.

Based on the above data, we propose that the heart-derived exosomes that circulate in the peripheral blood may be reporters of the myocardial injury, thus enabling for diagnosis and monitoring of cardiac patients. Consequently, we propose that blood exosome-based analyses should be further considered in the attempt to develop novel semi-invasive biomarkers of cardiac damage. Analyses of blood exosomes and exosomal miRs hold several properties that make them particularly interesting. From a mechanistic standpoint, exosomes are actively secreted from living cells[2, 4–6] and thus they might provide complementary information to markers, such as cTns and cardiac enzymes that mainly leak out of dying cardiomyocytes. Moreover, unlike the current biochemical biomarkers of myocardial injury, exosomes are not merely inert products: they reportedly elicit functional responses in recipient cells in a paracrine fashion and at distance [9–11, 13]. Consequently, exosomes could represent functional biomarkers directly involved in the development and progression of the pathological condition that we aim to diagnose or monitor. Analyses on left ventricle biopsies collected at different phases of cardiac surgeries may help validate the capacity of circulating exosomes to grade the level of myocardial injury. It is also important to investigate whether exosomes are elevated in the blood early after a MI and in other ischemic conditions resulting in high cTns level, such as pulmonary embolism and pulmonary arterial hypertension[26].

In this study, we have shown that the plasma concentration of exosome-sized nanoparticles is highly positively correlated with hs-cTn and that increases in plasma exosomes precede the appearance of detectable cTn in the blood after CABG. Hence, in the cardiac surgery and interventional cardiology settings, changes in the blood exosome concentration after a procedure could, *per se*, be a biomarker of the response of the patients to the intervention, possibly enabling prediction of clinical outcomes. This possibility requires validation in larger cohorts of patients. In case of a positive result, the measurement by NTA of the exosome-sized particle concentration in the blood could suffice. Of note, this method is relatively cheap and fast and requires an extremely small volume of biological fluid[2]. However, in most clinical scenarios, it might be mandatory to identify the cellular origin of the circulating exosomes. Moreover, while NTA is probably the best method currently available for quantifying exosomes, it is unable to unequivocally discriminate exosomes from non-vesicular nanoparticles of similar sizes. This represents a limitation of our study.

Exosomes are released by a variety of cell types and hence changes in their plasma concentration cannot be assumed to be dependent solely on their release from the heart cells. However, in the setting of cardiac surgery, the cardiomyocyte contribution to the circulating exosomes is strongly suggested by the presence of myocyte-enriched miRs (miR-1, miR-133a. miR-133b) in the plasma exosome cargo and in the fact that abundance of exosomal cardiac miRs increased after CABG.

Flow cytometry analyses have already been developed to understand the cellular origin of microparticles, which are bigger than exosomes[37]. Equivalent protocols helping the recognition of circulating exosomes with a cardiovascular origin are still lacking, especially because the small size of exosomes does not facilitate their analyses. In fact, for flow cytometry, exosomes can be bound to latex beads, but this impedes a precise quantification because the number of exosomes that are bound to each bead cannot be inferred[2, 38, 39]. However, novel, high-resolution flow cytometry analyses might overcome this obstacle[40]. ELISA has also been used to immobilize exosomes and allow for measuring exosome-enriched cancer biomarkers[41, 42]. The identification of cardiomyocyte-selective exosome antigens should facilitate the development of cardiac exosomes and their miR cargo as novel circulating biomarkers of myocardial injury. The Komuro group previously found exosomal miR-192 and miR-194 increased in the serum of patients with heart failure[43]. However, neither of these two miRs have been reported to be expressed by cardiomyocytes. Moreover, the study normalized the miR expression by U6[43], which is not a suitable endogenous control for the quantification of circulating miRs[44].

We did not observe any association between miR levels in whole plasma and hs-cTn-I in our CABG patients. Previous reports in the literature are inconsistent with respect to associations between individual cardiac-enriched miR concentrations in the whole plasma and cTns[17, 24, 45–47]. The translation of miRs to the diagnosis of ischemic myocardial damage needs additional work and at the moment it still looks unsuitable for obtaining quick responses guiding clinical decisions in surgery and interventional cardiology. For example, measurements of hs-cTns by immune-enzymatic reactions can be obtained in around 20 min, whereas PCR-based miR analyses are time-consuming and not completely standardized. Alternative techniques for miR quantification have been proposed,[48] but are not widely used.

We have found that after surgery, miR-24 and miR-210 were substantially enriched in the total pool of plasma exosomes. In contrast, the exosome/whole plasma concentration ratios of miR-1, miR-133a and miR-133b were mostly unchanged. This suggests the possibility that after CABG miR-24 and miR-210 are predominantly released *via* exosomes, while miR-1 and miR-133 are released *via* exosomes and exosome-independent mechanisms in similar proportions. The reasons beyond these differences cannot be inferred from the current data and require further investigation aiming to clarify the mechanisms governing exosome biogenesis (including exosome miR cargo formation) and secretion from the heart under homeostatic conditions and after a “cardiac stress”, such as ischemia/reperfusion.

In conclusion, the plasma concentrations of exosomes and their cargo of cardiac miRs change significantly in patients undergoing CABG. The circulating exosome numbers and their cargo of cardiac miRs are associated with hs-cTnI release. Moreover, the change in nanoparticle concentration precedes the cTn-I increase. Our findings suggest the trafficking of exosome-mediated miRs from the heart, which is amplified by cardiac surgery. Moreover, the data suggest a potential role for exosomes as new biomarkers of myocardial injury.

## Supporting Information

S1 Data Dataset used for analyses. (XLSX)

S1 Fig Changes in individual microRNA levels during CABG. (PDF)

S2 Fig Nanoparticle Tracking Analysis traces of the distribution of plasma particles from 6 randomly selected COPTIC CABG patients. (PDF)

S3 Fig Nanoparticle Tracking Analysis traces of the distribution of particles from each of the 6 ARCADIA patients. (PDF)

S4 Fig Cardiac troponin-I responses to CABG surgery. (PDF)

S1 Table List of microRNAs (miR) measured in the present study in the COPTIC and ARCADIA cohorts, with miR sequences and reasons for inclusion in the study. (PDF)

S2 Table Western Blot Antibodies. (PDF)

S3 Table Results of the tests of the effect of heparinase I treatment on RT-PCR cycles for an endogenous miR (miR-21) and of the spiked-in cel-miR-39 normalizer starting from heparinase I-treated and non-heparinase I-treated human plasma. (PDF)

S4 Table Mean values, and standard deviations (SD), of relative expression data for each microRNA. (PDF)

S5 Table High Sensitive cardiac troponin I (hs-cTn-I) measurements in consecut0069ve samples of the ARCADIA patients. (PDF)

## Abbreviations

CABG: coronary artery-by-pass graft surgery
CPB: cardiopulmonary by-pass
cTn: cardiac troponin
cTn-I: cardiac troponin I
EV: extracellular vesicle
MI: myocardial infarct
miR: microRNA
