# Supplementary material for: Coronary Artery-Bypass-Graft Surgery Increases the Plasma Concentration of Exosomes Carrying a Cargo of Cardiac MicroRNAs: An Example of Exosome Trafficking Out of the Human Heart with Potential for Cardiac Biomarker Discovery
Source: PLoS One. 2016 Apr 29;11(4):e0154274. doi: 10.1371/journal.pone.0154274 (PMC4851293; doi:10.1371/journal.pone.0154274)
Supplement: S1 Fig — (PDF) [file pone.0154274.s002.pdf]

Supplemental Figure 1

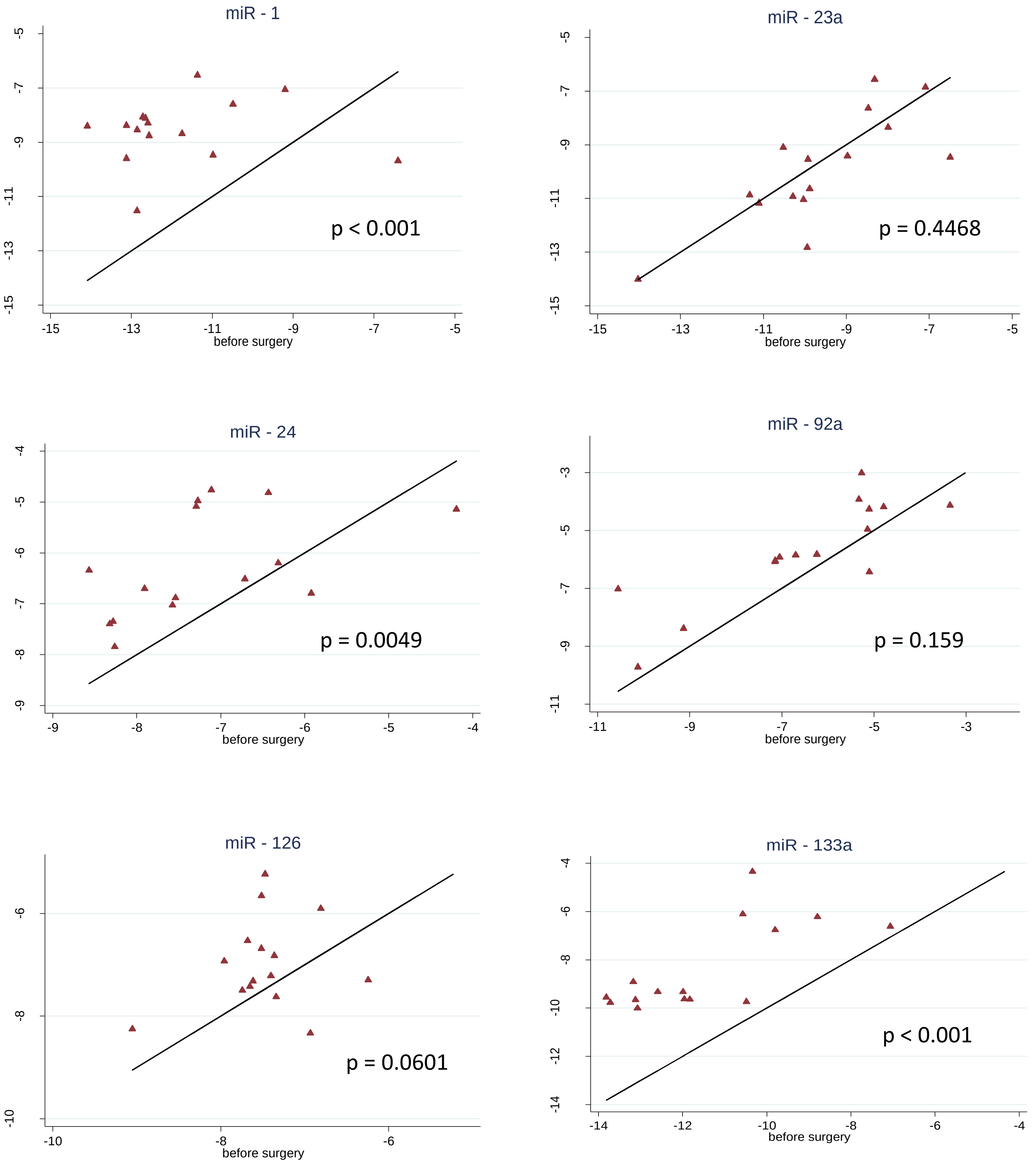

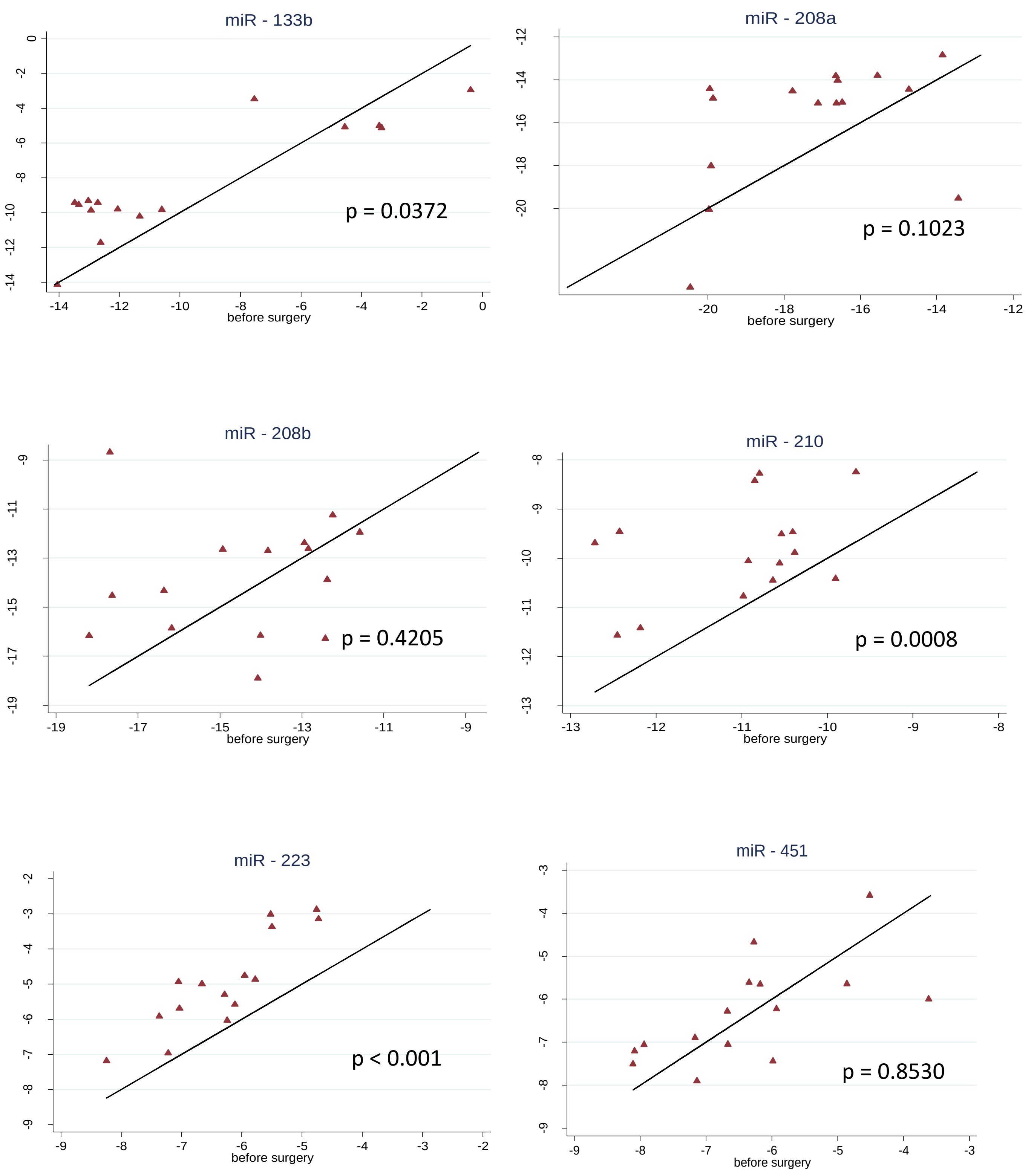

<u>Supplemental Figure 1</u>: Changes in individual microRNA levels during CABG. MicroRNAs (miRs) were measured in the plasma obtained before and early at termination of surgery in 15 CABG patients recruited for the COPTIC biobank. The graphs show pre- vs. post-operation levels of the individual miRs (the solid line represents no change between the pre- and post-operation levels). Data were analysed by linear regression. The individual P values are presented on each graph.
