# Supplementary material for: Coronary Artery-Bypass-Graft Surgery Increases the Plasma Concentration of Exosomes Carrying a Cargo of Cardiac MicroRNAs: An Example of Exosome Trafficking Out of the Human Heart with Potential for Cardiac Biomarker Discovery
Source: PLoS One. 2016 Apr 29;11(4):e0154274. doi: 10.1371/journal.pone.0154274 (PMC4851293; doi:10.1371/journal.pone.0154274)
Supplement: S2 Fig — (PDF) [file pone.0154274.s003.pdf]

Supplemental Figure 2

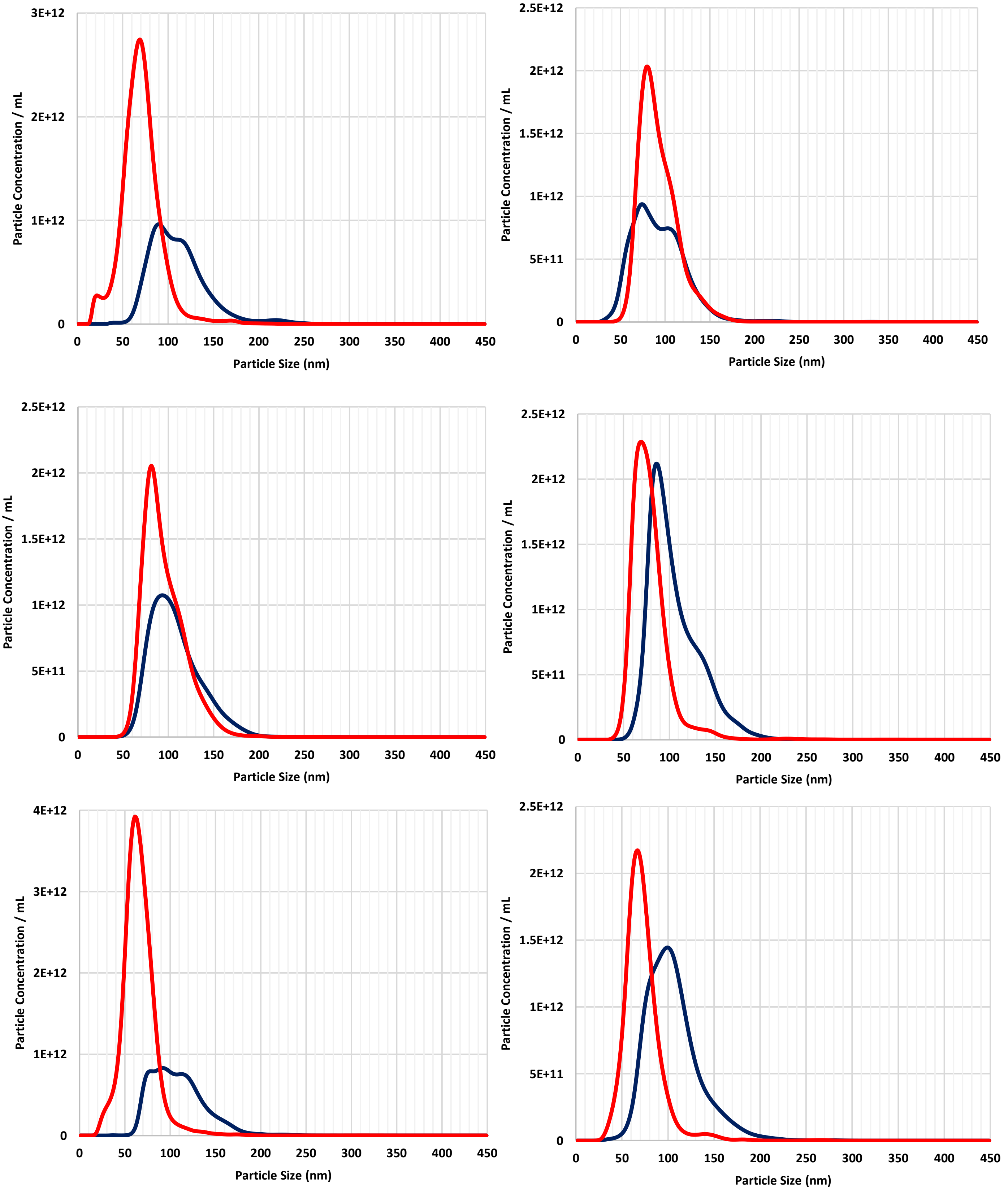

<u>Supplemental Figure 2</u>: Nanoparticle Tracking Analysis traces of the distribution of plasma particles from 6 randomly selected COPTIC CABG patients. The graphs show the pre-operative and post-operative particle size distributions as determined by Nanoparticle Tracking Analysis. Each patient shows a considerable increase in the plasma concentration of particles of exosomal size (30 to 100 nm) following surgery. Blue line – pre-operative size distribution, red line – post-operative size distribution.
