# Supplementary material for: Coronary Artery-Bypass-Graft Surgery Increases the Plasma Concentration of Exosomes Carrying a Cargo of Cardiac MicroRNAs: An Example of Exosome Trafficking Out of the Human Heart with Potential for Cardiac Biomarker Discovery
Source: PLoS One. 2016 Apr 29;11(4):e0154274. doi: 10.1371/journal.pone.0154274 (PMC4851293; doi:10.1371/journal.pone.0154274)
Supplement: S3 Fig — (PDF) [file pone.0154274.s004.pdf]

Supplemental Figure 3

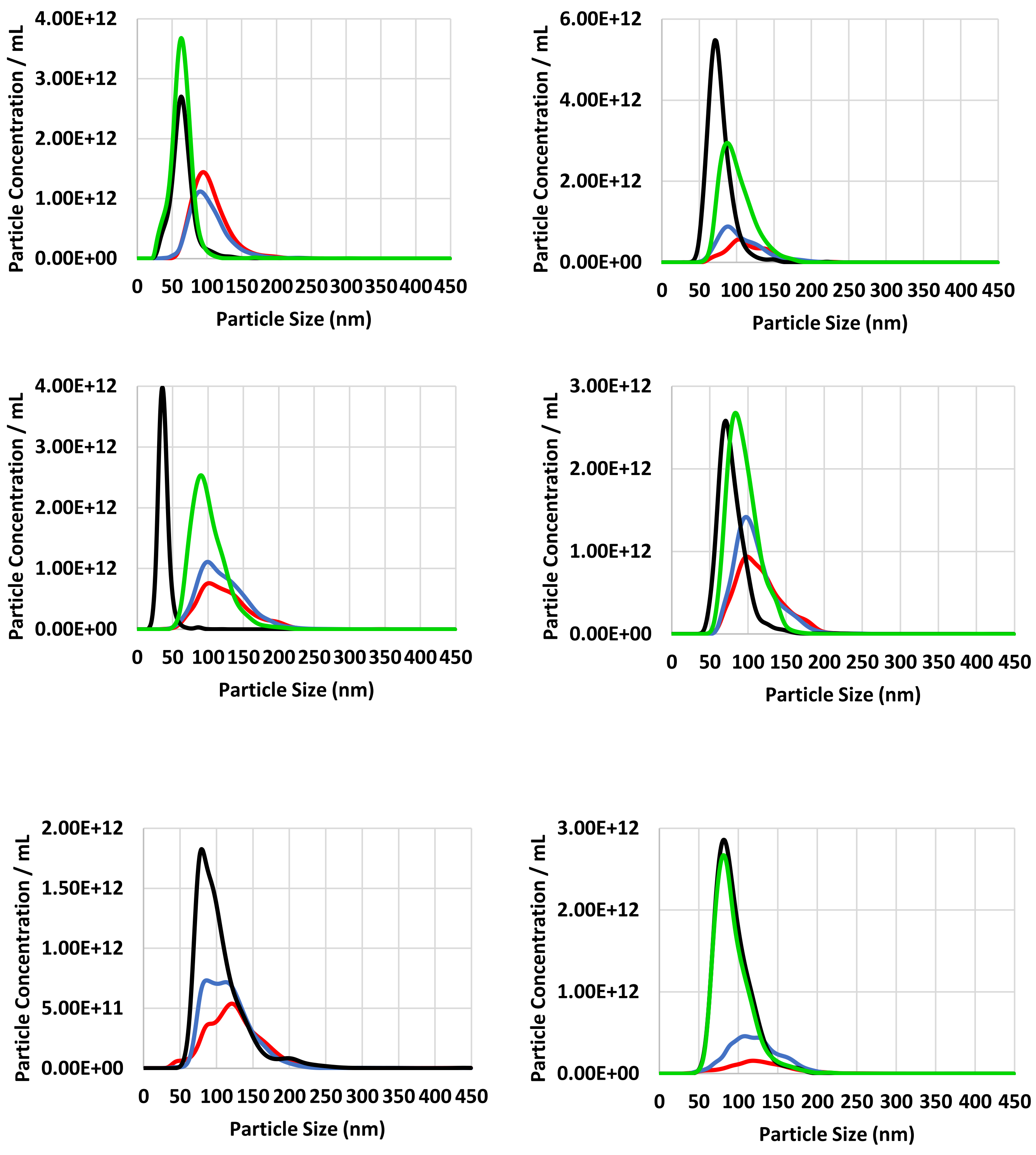

Supplemental Figure 3: Nanoparticle Tracking Analysis traces of the distribution of particles from each of the 6 ARCADIA patients. Each patient shows a considerable increase in particles of exosomal size following surgery. Red line: before the operation; blue line: during the operation, before initiation of CPB; black line: 24 hours post-surgery; Green line: 48 hours post-surgery. The 48h post-operation sample for one patient (E) was missing
