# Supplementary material for: Coronary Artery-Bypass-Graft Surgery Increases the Plasma Concentration of Exosomes Carrying a Cargo of Cardiac MicroRNAs: An Example of Exosome Trafficking Out of the Human Heart with Potential for Cardiac Biomarker Discovery
Source: PLoS One. 2016 Apr 29;11(4):e0154274. doi: 10.1371/journal.pone.0154274 (PMC4851293; doi:10.1371/journal.pone.0154274)
Supplement: S4 Fig — (PDF) [file pone.0154274.s005.pdf]

Supplemental Figure 4

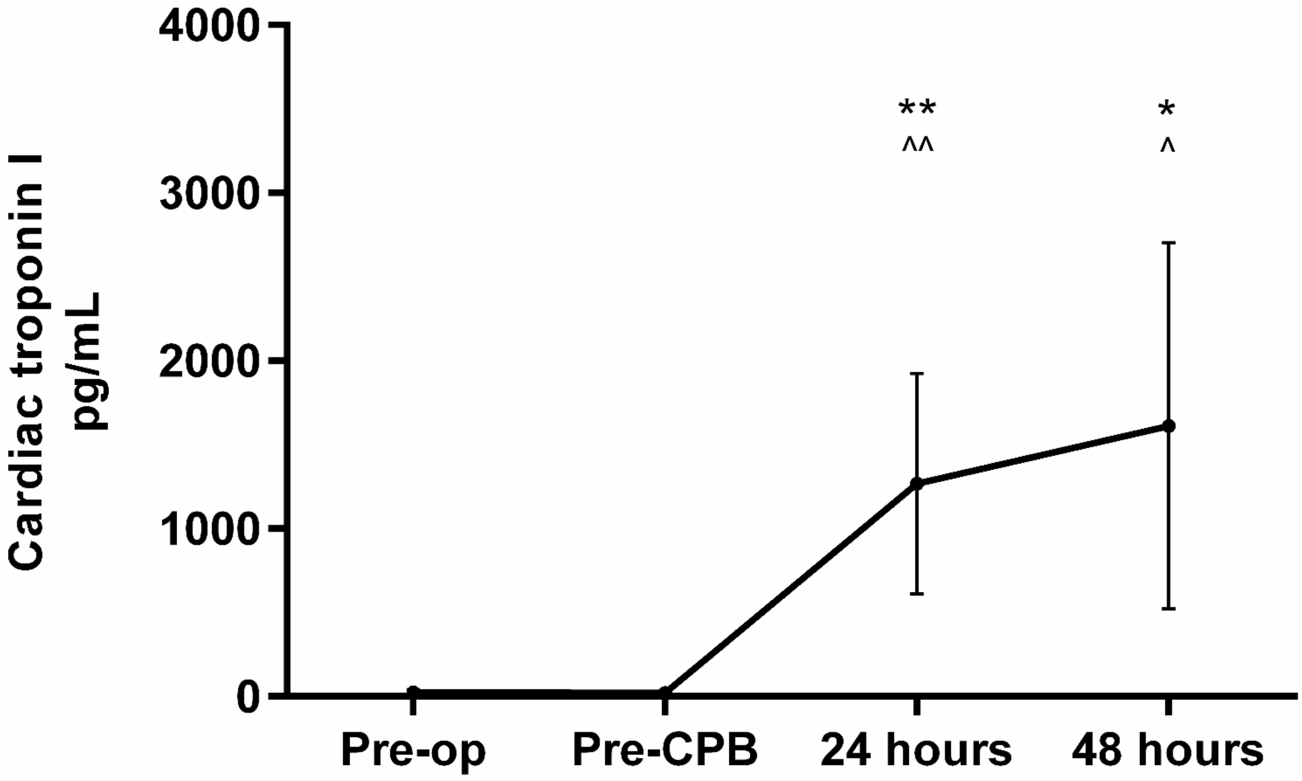

<u>Supplemental Figure 4</u> – **Cardiac troponin-I responses to CABG surgery.** Time course of cardiac troponin I levels in the ARCADIA study samples: plasma concentrations of cardiac troponin I in the serial plasma samples (n=6). Plasma was prepared from blood collected immediately before (pre-operation, pre-op), during the operation before (pre-CPB) starting coronary grafting with the patient on-CPB and at 24h and 48h post-completion of surgery. Data are shown as mean ± SEM. * $p < 0.05$, ** $p < 0.01$ *vs.* pre-op; ^ $p < 0.05$, ^^ $p < 0.01$ *vs.* pre-CPB; repeated measures ANOVA with *post hoc* Tukey's test on log-transformed data.
