# Supplementary material for: Coronary Artery-Bypass-Graft Surgery Increases the Plasma Concentration of Exosomes Carrying a Cargo of Cardiac MicroRNAs: An Example of Exosome Trafficking Out of the Human Heart with Potential for Cardiac Biomarker Discovery
Source: PLoS One. 2016 Apr 29;11(4):e0154274. doi: 10.1371/journal.pone.0154274 (PMC4851293; doi:10.1371/journal.pone.0154274)
Supplement: S1 Table — (PDF) [file pone.0154274.s006.pdf]

Supplemental Table 1: List of microRNAs (miR) measured in the present study in the COPTIC and ARCADIA cohorts, with miR sequences and reasons for inclusion in the study.

microRNA Base ID	Reasons for inclusion	COPTIC (plasma)	ARCADIA (plasma and exosome)	Stem-loop Sequence (5'-3'), including the mature form sequence (in red)	Catalogue number (Life technologies Ltd)
hsa-miR-1-3p	Enriched in muscles; Increased in peripheral blood after MI, open heart surgery and trans-coronary ablation of septal hypertrophy. [1-7]	Y	Y	5'-UGGGAAACAUACUUCUUUAUAUGCCCAUAUGGACCUGCUAAGCUA**UGGAAUGUAAAGAAGUAUGUAU**CUCA-3'	002222
hsa-miR-23a-3p	Control: Supposedly stable expressed in plasma.[8]	Y		5'-GGCCGGCUGGGGUUCCUGGGGAUGGGAUUUGCUUCCUGUCACAA**AUCACAUUGCCAGGGAUUUCCA**ACCGACC-3'	000399
hsa-miR-24-3p	Expressed in the mouse myocardium, where it is increased after a MI, but it is reduced by MI in cardiac myocytes, suggesting their extracellular release of miR-24.[9]	Y	Y	5'-CUCCGGUGCCUACUGAGCUGAUAUCAGUUCUCAUUUUACACAC**UGGCUCAGUUCAGCAGGAACAG**GAG-3'	000402
hsa-miR -92a-3p	Expressed in the vasculature and in increased by ischemia in the mouse and pig ischemic heart.[10, 11]	Y		5'-CUUUCUACACAGGUUGGGAUCGGUUGCAAUGCUGUGUUUCUG**UAUGGUAUUGCACUUGUCCCGGCCUGU**UGAGUUUGG-3'	000431
hsa-miR-122-5p	Control: Highly enriched in liver and supposedly not expressed in the heart[12]		Y	5'-CCUUAGCAGAGCUG**UGGAGUGUGACAAUGGUGUUUG**UGUCUAAACUAUCAAACGCCAUUAUCACACUAAAUAGCUACUGCUAGGC-3'	002245
hsa-miR-126-3p	Expressed by endothelial cells and platelets.[13, 14]	Y		5'-CGGCCCAUUAUUACUUUUGGUACGCGCUAUGCCACUCUCAAC**UCGUACCGUGAGUAAUAAUGC**-3'	000451
hsa-miR-133a-3p	Enriched in muscles and increased in the blood of patients with myocardial infarction and transcoronary ablation of septal hypertrophy.[5, 6 7, 15]	Y	Y	5'-ACAAUGCUUUGCUAGAGCUGGUAAAAUGGAACCAAAUCGCCUCUUCAAUGGAU**UUGGUCCCCUUCAACCAGCUG**UAGCUAUGCAUUGA-3'	002246
hsa-miR -133b	Enriched in muscles and increased in the blood of patients with myocardial infarct.[1, 6]	Y	Y	5'-UUGAUUGGACAAGGUAUGCUAUGACGGACAUUUACAUACCUGGUUGUAGUCGAACCAAAUUGUUAUAUUUUUAAAAUCAA**UUGGUCCCCUUCAACCAGCUA**UGUUUCUCCUCCUGUAAACAUCUAGUUAA-3'	002247
hsa-miR-208a-3p	Enriched in cardiac myocytes; Increased in the blood of patients with either a myocardial infarct or receiving trans-coronary ablation of septal hypertrophy.[1, 6]	Y		5'-UGACGGGCGAGCUUUUGGCCCGGGUUAUACCUGAUGCUCACGU**AUAAGACGAGCAAAAAGCUUGU**UGGUCA-3'	000511
hsa-miR-208b-3p	Enriched in cardiac myocytes; It increases in the blood of patients with a myocardial infarct.[6, 7]	Y		5'-CCUCUCAGGGAAGCUUUUUGCUCGAAUUAUGUUUCUGAUCCGAAUA**UAAGACGA**	002290

				**ACAAAAGGUUUGU**CUGAGGGCAG-3’	
hsa-miR-210-3p	“Hypoxia microRNA”:<br>It increases under hypoxic condition, including in cardiac myocytes. It is expressed in the heart.[16, 17]	Y	Y	5’-ACCCGGCAGUGCCUCCAGGCGCAGGGCAGCCCCUGCCCACCGCACACUGCGCUGCCCCAGACCCA**CUGUGCGUGUGACAGCGGCUGA**UCUGUGCCUGGGCAGCGCGACCC-3’	000512
hsa-miR-223-3p	Expressed in platelets; it increases in the blood following platelets activation.[14]	Y		5’-CCUGGCCUCCUGCAGUGCCACGCUCCGUGUAUUUGACAAGCUGAGUUGGACACUCCAUGUGGUAGAG**UGUCAGUUUGUCAAAUACCCCA**AGUGCGGCACAUGCUUACCAG-3’	002295
hsa-miR-451	Control:<br>measured as a quality control against haemolysis since it is enriched in red blood cells.[18]	Y		5’-CUUGGGAAUGGCAAGG**AAACCGUUACCAUUACUGAGUU**UAGUAAUGGUAAUGGUUCUCUUGCUAUACCCAGA-3’	001141
cel-miR-39-3p	Control:<br>Spike in control used to normalize has-miRs expressional data.[1, 3, 5, 6, 19, 20]	Y	Y	5’-UAUACCGAGAGCCCAGCUGAUUUCGUCUUGGUAAUAAGCUCGUCAUUGAGAUUA**UCACCGGGUGUAAAUCAGCUUG**GCUCUGGUGUC-3’	000200

References

1. Liebetrau C, Mollmann H, Dorr O, Szardien S, Troidl C, Willmer M, Voss S, Gaede L, Rixe J, Rolf A, Hamm C and Nef H. Release kinetics of circulating muscle-enriched microRNAs in patients undergoing transcoronary ablation of septal hypertrophy. *Journal of the American College of Cardiology*. 2013;62:992-8.
2. Zhou X, Mao A, Wang X, Duan X, Yao Y and Zhang C. Urine and serum microRNA-1 as novel biomarkers for myocardial injury in open-heart surgeries with cardiopulmonary bypass. *PloS one*. 2013;8:e62245.
3. D'Alessandra Y, Devanna P, Limana F, Straino S, Di Carlo A, Brambilla PG, Rubino M, Carena MC, Spazzafumo L, De Simone M, Micheli B, Biglioli P, Achilli F, Martelli F, Maggiolini S, Marenzi G, Pompilio G and Capogrossi MC. Circulating microRNAs are new and sensitive biomarkers of myocardial infarction. *European heart journal*. 2010;31:2765-73.
4. Yang W, Shao J, Bai X and Zhang G. Expression of Plasma microRNA-1/21/ 208a/499 in Myocardial Ischemic Reperfusion Injury. *Cardiology*. 2015;130:237-241.
5. Wang GK, Zhu JQ, Zhang JT, Li Q, Li Y, He J, Qin YW and Jing Q. Circulating microRNA: a novel potential biomarker for early diagnosis of acute myocardial infarction in humans. *European heart journal*. 2010;31:659-66.
6. Widera C, Gupta SK, Lorenzen JM, Bang C, Bauersachs J, Bethmann K, Kempf T, Wollert KC and Thum T. Diagnostic and prognostic impact of six circulating microRNAs in acute coronary syndrome. *Journal of molecular and cellular cardiology*. 2011;51:872-5.
7. Gidlof O, Andersson P, van der Pals J, Gotberg M and Erlinge D. Cardiospecific microRNA plasma levels correlate with troponin and cardiac function in patients with ST elevation myocardial infarction, are selectively dependent on renal elimination, and can be detected in urine samples. *Cardiology*. 2011;118:217-26.
8. Blondal T, Jensby Nielsen S, Baker A, Andreasen D, Mouritzen P, Wrang Teilum M and Dahlsveen IK. Assessing sample and miRNA profile quality in serum and plasma or other biofluids. *Methods*. 2013;59:S1-6.
9. Meloni M, Marchetti M, Garner K, Littlejohns B, Sala-Newby G, Xenophontos N, Floris I, Suleiman MS, Madeddu P, Caporali A and Emanueli C. Local inhibition of microRNA-24 improves reparative angiogenesis and left ventricle remodeling and function in mice with myocardial infarction. *Molecular therapy : the journal of the American Society of Gene Therapy*. 2013;21:1390-402.
10. Bonauer A, Carmona G, Iwasaki M, Mione M, Koyanagi M, Fischer A, Burchfield J, Fox H, Doebele C, Ohtani K, Chavakis E, Potente M, Tjwa M, Urbich C, Zeiher AM and Dimmeler S. MicroRNA-92a controls angiogenesis and functional recovery of ischemic tissues in mice. *Science*. 2009;324:1710-3.
11. Hinkel R, Penzkofer D, Zuhlke S, Fischer A, Husada W, Xu QF, Baloch E, van Rooij E, Zeiher AM, Kupatt C and Dimmeler S. Inhibition of microRNA-92a protects against ischemia/reperfusion injury in a large-animal model. *Circulation*. 2013;128:1066-75.
12. Chang J, Provost P and Taylor JM. Resistance of human hepatitis delta virus RNAs to dicer activity. *Journal of virology*. 2003;77:11910-7.
13. Kuehbacher A, Urbich C, Zeiher AM and Dimmeler S. Role of Dicer and Drosha for endothelial microRNA expression and angiogenesis. *Circulation research*. 2007;101:59-68.
14. Willeit P, Zampetaki A, Dudek K, Kaudewitz D, King A, Kirkby NS, Crosby-Nwaobi R, Prokopi M, Drozdov I, Langley SR, Sivaprasad S, Markus HS, Mitchell JA, Warner TD, Kiechl S and Mayr M. Circulating microRNAs as novel biomarkers for platelet activation. *Circulation research*. 2013;112:595-600.
15. Kuwabara Y, Ono K, Horie T, Nishi H, Nagao K, Kinoshita M, Watanabe S, Baba O, Kojima Y, Shizuta S, Imai M, Tamura T, Kita T and Kimura T. Increased microRNA-1 and microRNA-133a levels in serum of patients with cardiovascular disease indicate myocardial damage. *Circulation Cardiovascular genetics*. 2011;4:446-54.
16. Mutharasan RK, Nagpal V, Ichikawa Y and Ardehali H. microRNA-210 is upregulated in hypoxic cardiomyocytes through Akt- and p53-dependent pathways and exerts cytoprotective effects. *American journal of physiology Heart and circulatory physiology*. 2011;301:H1519-30.
17. Chan YC, Banerjee J, Choi SY and Sen CK. miR-210: the master hypoxamir. *Microcirculation*. 2012;19:215-23.

18. Kirschner MB, Kao SC, Edelman JJ, Armstrong NJ, Vallely MP, van Zandwijk N and Reid G. Haemolysis during sample preparation alters microRNA content of plasma. *PloS one*. 2011;6:e24145.
19. Spinetti G, Fortunato O, Caporali A, Shantikumar S, Marchetti M, Meloni M, Descamps B, Floris I, Sangalli E, Vono R, Faglia E, Specchia C, Pintus G, Madeddu P and Emanueli C. MicroRNA-15a and microRNA-16 impair human circulating proangiogenic cell functions and are increased in the proangiogenic cells and serum of patients with critical limb ischemia. *Circulation research*. 2013;112:335-46.
20. Eitel I, Adams V, Dieterich P, Fuernau G, de Waha S, Desch S, Schuler G and Thiele H. Relation of circulating MicroRNA-133a concentrations with myocardial damage and clinical prognosis in ST-elevation myocardial infarction. *American heart journal*. 2012;164:706-14.
