# Supplementary material for: Coronary Artery-Bypass-Graft Surgery Increases the Plasma Concentration of Exosomes Carrying a Cargo of Cardiac MicroRNAs: An Example of Exosome Trafficking Out of the Human Heart with Potential for Cardiac Biomarker Discovery
Source: PLoS One. 2016 Apr 29;11(4):e0154274. doi: 10.1371/journal.pone.0154274 (PMC4851293; doi:10.1371/journal.pone.0154274)
Supplement: S2 Table — (PDF) [file pone.0154274.s007.pdf]

Supplemental Table 2: Western Blot Antibodies.

Antibody	Supplier	Size (kDa)	host	Antibody	Cat Number	Dilution
Alix	Millipore	75	Rabbit	Primary Polyclonal	ABC40	1:1,000
Flotillin-1	BD	48	Mouse	Primary Monoclonal	A610820	1:1,000
EpCAM	Cell Signaling	40	Rabbit	Primary Monoclonal	2626	1:1,000
CD63	Abcam	26	Mouse	Primary Monoclonal	ab59479	1:1,000
ECL Mouse IgG	GE Healthcare		Sheep	Secondary	NA931	1:2,000
ECL Mouse IgG	GE Healthcare		Donkey	Secondary	NA934	1:2,000
