# Supplementary material for: Coronary Artery-Bypass-Graft Surgery Increases the Plasma Concentration of Exosomes Carrying a Cargo of Cardiac MicroRNAs: An Example of Exosome Trafficking Out of the Human Heart with Potential for Cardiac Biomarker Discovery
Source: PLoS One. 2016 Apr 29;11(4):e0154274. doi: 10.1371/journal.pone.0154274 (PMC4851293; doi:10.1371/journal.pone.0154274)
Supplement: S3 Table — (PDF) [file pone.0154274.s008.pdf]

<u>Supplemental Table 3</u>: Results of the tests of the effect of heparinase I treatment on RT-PCR cycles for an endogenous miR (miR-21) and of the spiked-in cel-miR-39 normalizer starting from heparinase I-treated and non-heparinase I-treated human plasma.

A. Example of the effect of Heparinase I treatment in plasma prepared from blood taken before anticoagulation treatment.

**Heparinase I**	**-**	**+**
**MicroRNA**	**Ct Values**	**Ct Values**
**cel-miR-39**	18.49	19.01
	18.59	19.00
	18.73	18.88
**hsa-miR-21**	30.30	30.63
	31.08	30.82
	29.98	30.22

B. Example of the effect of Heparinase I treatment in plasma prepared from blood taken during heparin treatment.

**Heparinase I**	**-**	**+**
**MicroRNA**	**Ct Values**	**Ct Values**
**cel-miR-39**	25.34	18.45
	25.41	18.30
	25.67	18.45
**hsa-miR-21**	37.31	30.14
	37.59	30.04
	37.44	29.76
