# Supplementary material for: Coronary Artery-Bypass-Graft Surgery Increases the Plasma Concentration of Exosomes Carrying a Cargo of Cardiac MicroRNAs: An Example of Exosome Trafficking Out of the Human Heart with Potential for Cardiac Biomarker Discovery
Source: PLoS One. 2016 Apr 29;11(4):e0154274. doi: 10.1371/journal.pone.0154274 (PMC4851293; doi:10.1371/journal.pone.0154274)
Supplement: S4 Table — (PDF) [file pone.0154274.s009.pdf]

Supplemental Table 4 Mean values, and standard deviations (SD), of relative expression data for each microRNA

Table A: Supplemental to Figure 3. Mean values, and standard deviations (SD), of relative expression data for each microRNA in the whole ARCADIA plasma. MicroRNA expression was calculated relative to exogenous cel-miR-39, using the formula $2^{-[(\text{miR CT}) - (\text{cel-miR-39 CT})]}$.

**microRNAs (miRs)**		**Average relative expression (SD)**			
		**Pre-op**	**Pre-CPB**	**24 hours**	**48 hours**
**miR-1**	x10-4	1.14 (0.62)	2.31 (0.63)	5.00 (2.70)	1.51 (0.93)
**miR-24**	x10-2	1.72 (0.87)	1.45 (0.36)	0.79 (0.19)	1.80 (1.33)
**miR-133a**	x10-4	6.46 (4.86)	10.13 (5.82)	15.46 (9.68)	5.39 (1.04)
**miR-133b**	x10-4	4.32 (1.71)	7.54 (2.44)	12.70 (6.38)	5.36 (2.17)
**miR-210**	x10-4	4.00 (3.36)	2.76 (1.46)	2.40 (1.65)	3.88 (1.44)
**miR-122**	x10-2	5.50 (3.04)	8.00 (5.49)	2.23 (2.97)	2.04 (1.45)

Pre-op: pre-operation (in the anaesthetic room); pre-CPB: during surgery before establishment of the cardiopulmonary by-pass (CPB); 24 hours: 24 hours after end of CBP; 48 hours: 48 hours after end of CBP.

Table B: Supplemental to Figure 4C. Mean values, and standard deviations (SD), of relative expression data for each microRNA in the ARCADIA plasma exosomal fraction. MicroRNA expression was calculated relative to exogenous cel-miR-39, using the formula $2^{-[(\text{miR CT}) – (\text{cel-miR-39 CT})]}$.

microRNAs		Average relative expression (SD)			
(miRs)		Pre-op	Pre-CPB	24 hours	48 hours
**miR-1**	x10-7	3.76 (0.62)	4.88 (1.54)	9.20 (2.45)	9.78 (2.63)
**miR-24**	x10-7	51.51 (14.62)	75.90 (15.71)	135.69 (23.94)	116.89 (34.52)
**miR-133a**	x10-7	5.31 (2.11)	4.77 (1.50)	15.15 (2.80)	13.16 (4.41)
**miR-133b**	x10-7	3.86 (1.39)	5.93 (3.60)	15.64 (4.39)	8.79 (3.57)
**miR-210**	x10-7	0.80 (0.17)	0.89 (0.25)	1.57 (0.31)	1.41 (0.72)
**miR-122**	x10-7	21.61 (3.26)	19.32 (3.84)	13.82 (4.50)	8.47 (2.57)

Pre-op: pre-operation (in the anaesthetic room); pre-CPB: during surgery before establishment of the cardiopulmonary by-pass (CPB); 24 hours: 24 hours after end of CBP; 48 hours: 48 hours after end of CBP.
