# Supplementary material for: Coronary Artery-Bypass-Graft Surgery Increases the Plasma Concentration of Exosomes Carrying a Cargo of Cardiac MicroRNAs: An Example of Exosome Trafficking Out of the Human Heart with Potential for Cardiac Biomarker Discovery
Source: PLoS One. 2016 Apr 29;11(4):e0154274. doi: 10.1371/journal.pone.0154274 (PMC4851293; doi:10.1371/journal.pone.0154274)
Supplement: S5 Table — (PDF) [file pone.0154274.s010.pdf]

**<u>Supplemental Table 5</u>: High Sensitive cardiac troponin I (hs-cTn-I) measurements in consecutive samples of the ARCADIA patients**

| **Patient** | **hs-cTn-I values (pg/mL)** | | | |
|---|---|---|---|---|
| | **Pre-op** | **Pre-CPB** | **24 hours post-CPB** | **48 hours post-CPB** |
| **1** | 14.4 | 9.7 | 4335.8 | 6914.6 |
| **2** | 1.4 | 2.0 | 236.0 | 244.7 |
| **3** | 12.4 | 9.8 | 346.0 | 152.3 |
| **4** | 2.0 | 2.2 | 279.5 | 207.9 |
| **5** | 110.2 | 98.8 | 1772.4 | * |
| **6** | 3.5 | 2.9 | 638.1 | 369.6 |

***** The plasma sample for this time point was unavailable.

Serial plasma cardiac troponin I values in the individual patients from the ARCADIA study set, measured using a high-sensitivity assay. Samples were collected immediately before the operation (pre-op), during the operation before initiation of cardiopulmonary bypass (pre-CPB), and at 24h and 48h post-operatively. All values are given in pg/mL.
